# Supplementary material for: Factors predicting parenting stress in the autism spectrum disorder context: A network analysis approach
Source: PLoS One. 2025 Apr 21;20(4):e0319036. doi: 10.1371/journal.pone.0319036 (PMC12011239; doi:10.1371/journal.pone.0319036)
Supplement: S1 File — Figure S1. Hybrid Bayesian network without blacklist and excluding some demographics. Figure S2. Hybrid Bayesian network with blacklist and including all demographics. Figure S3. Hybrid Bayesian network without blacklist and including all demographics. Figure S4. Averaged hybrid Bayesian network with blacklist excluding some demographics. Figure S5. Path analysis of Hybrid Bayesian network without blacklist with excluding some demographics. Figure S6. Path analysis of hybrid Bayesian network with blacklist with all demographics. Figure S7. Path analysis of hybrid Bayesian network without blacklist with all demographics. Figure S8. Centrality statistics Bayesian network with blacklist excluding some demographics. Table S1. Centrality statistics variable codes. (DOCX) [file pone.0319036.s001.docx]

**Supplementary Information**

**Figure 1**

*Hybrid Bayesian Network Without Blacklist and excluding some Demographics*

*
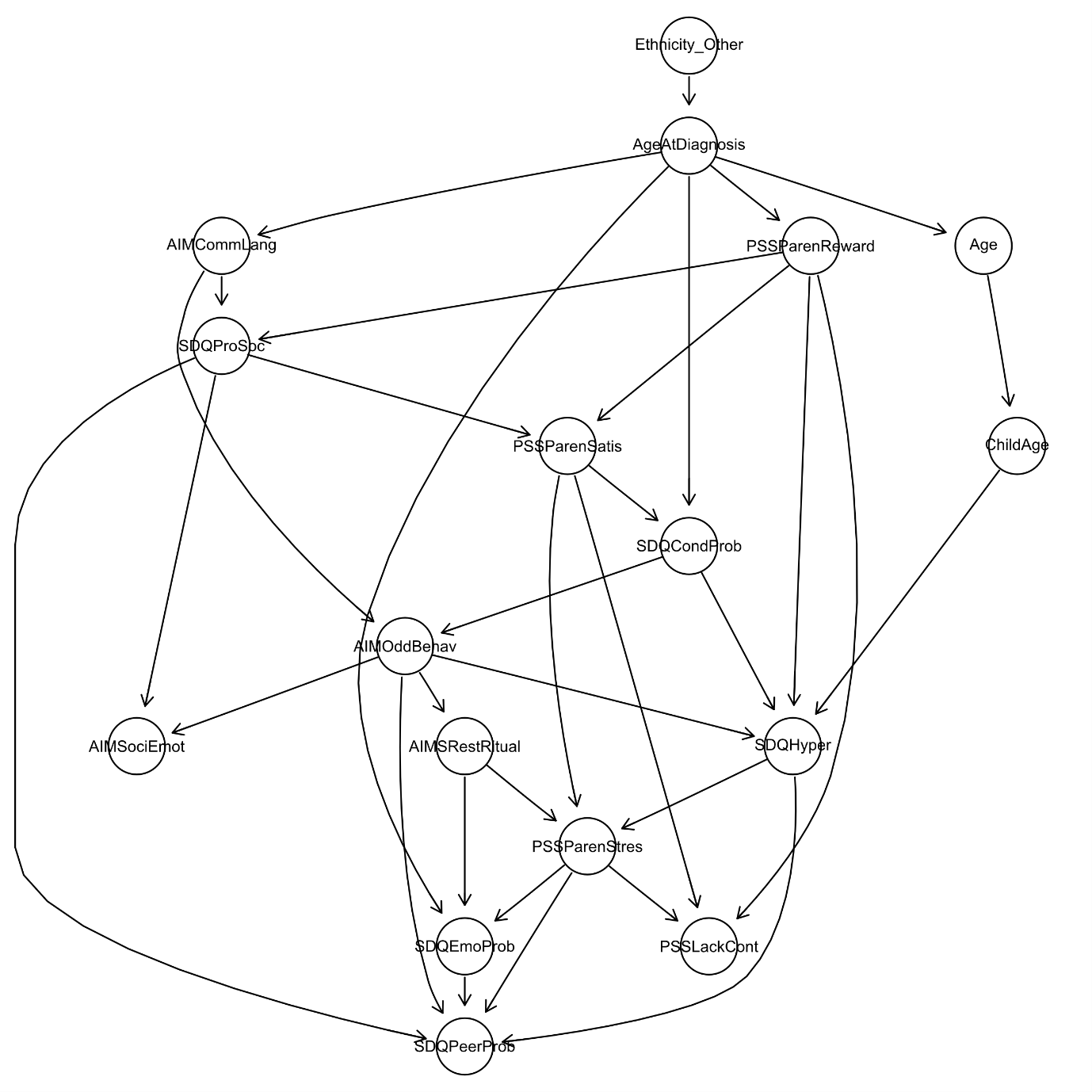
*

**Figure 2**

*Hybrid Bayesian Network With Blacklist and including all Demographics*

*
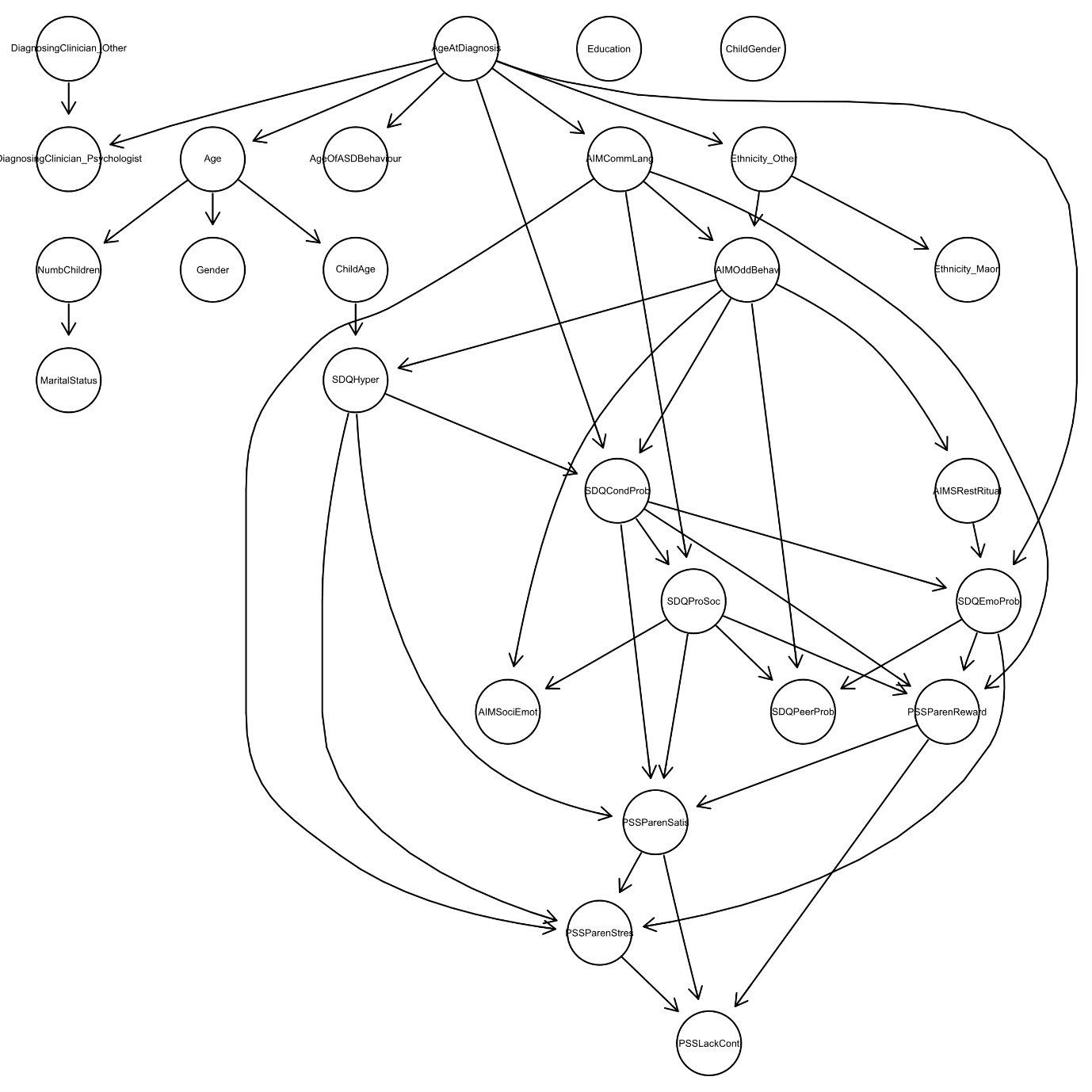
*

**Figure 3**

*Hybrid Bayesian Network Without Blacklist and including all Demographics*


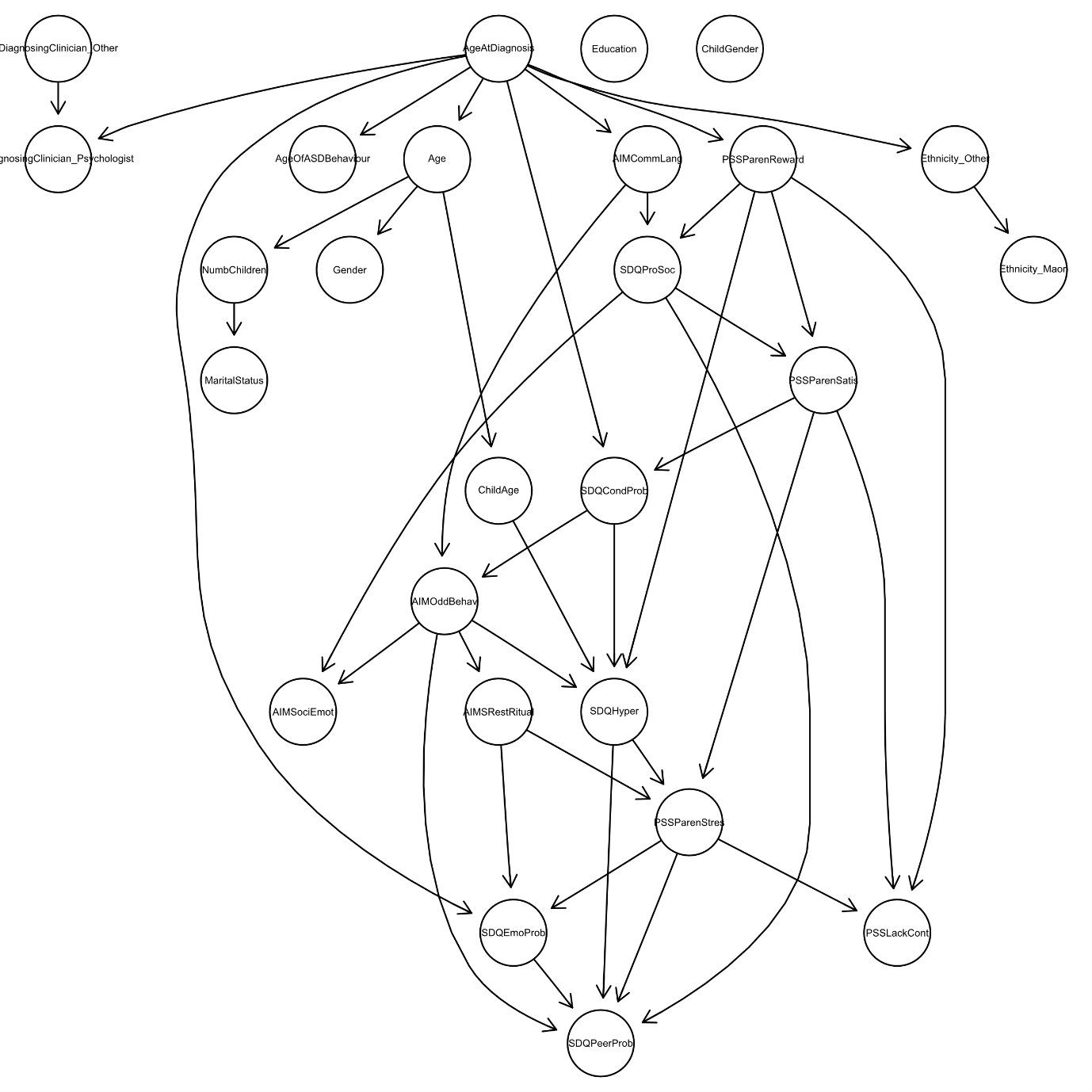


**Figure 4**

*Averaged Hybrid Bayesian Network With Blacklist Excluding Some Demographics*


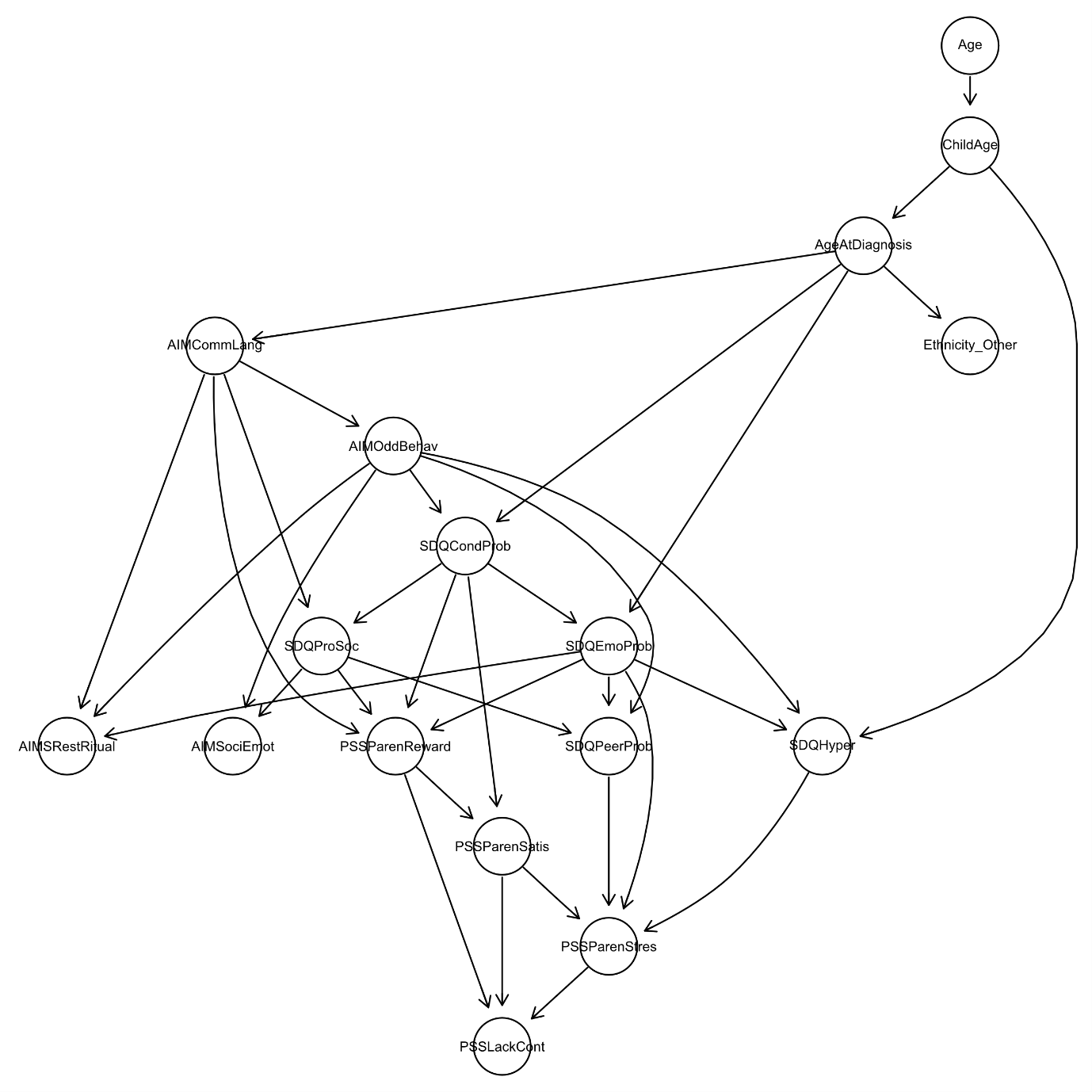


**Figure 5**

*Path Analysis of Hybrid Bayesian Network without Blacklist with excluding some demographics*


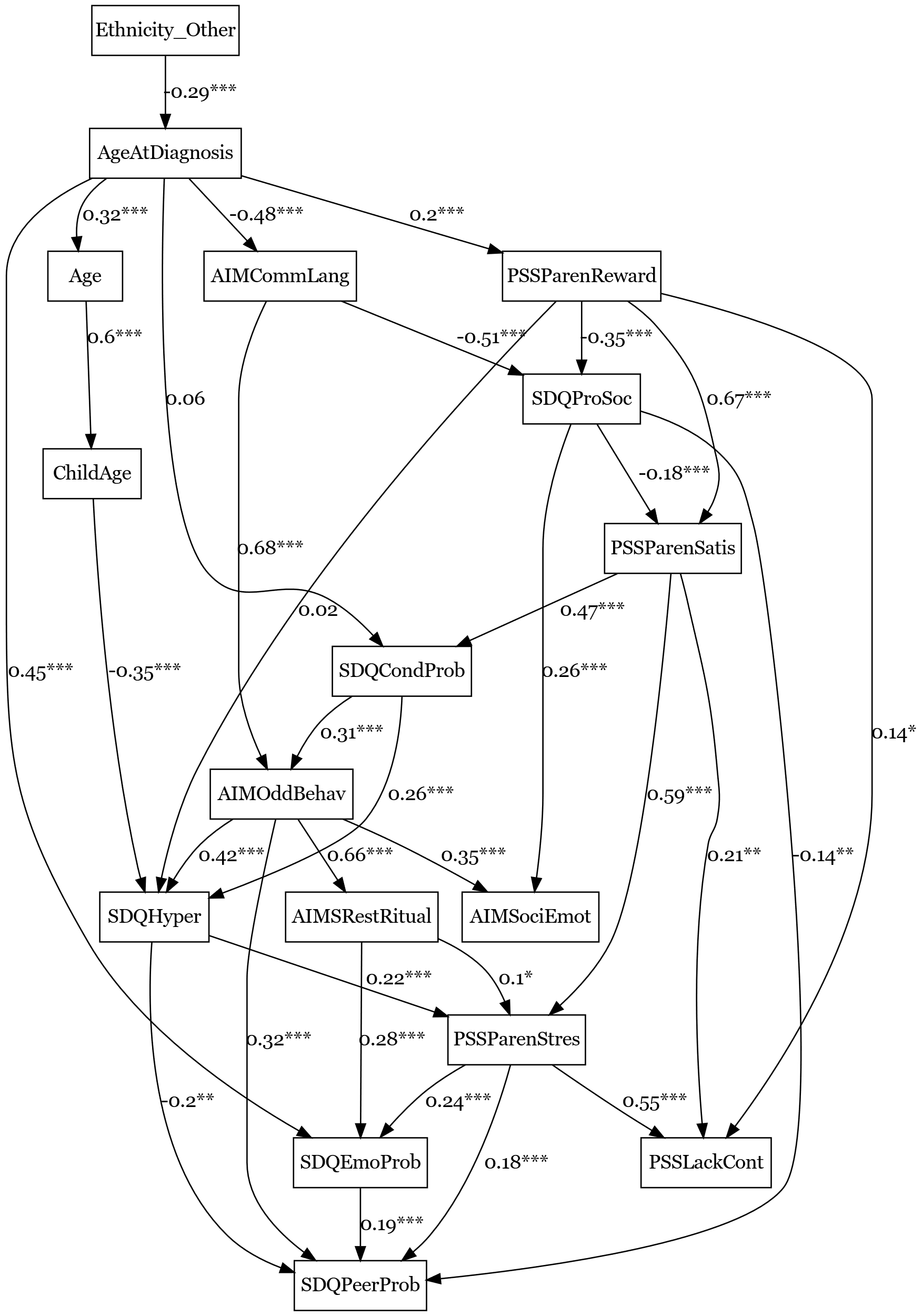


**Figure 6**

*Path Analysis of Hybrid Bayesian Network with Blacklist with all demographics*

**
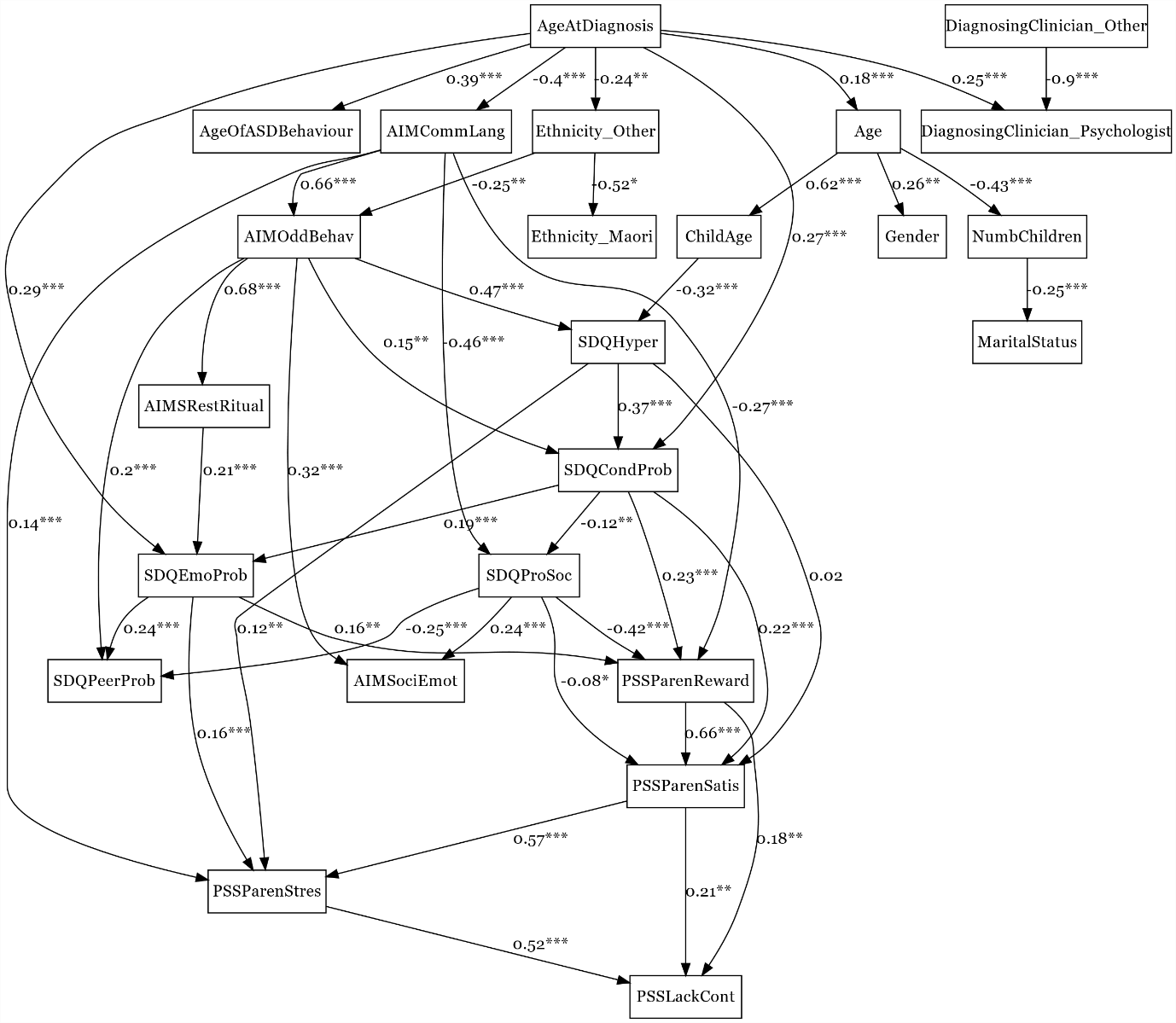
**

**Figure 7**

*Path Analysis of Hybrid Bayesian Network without Blacklist with all demographics*


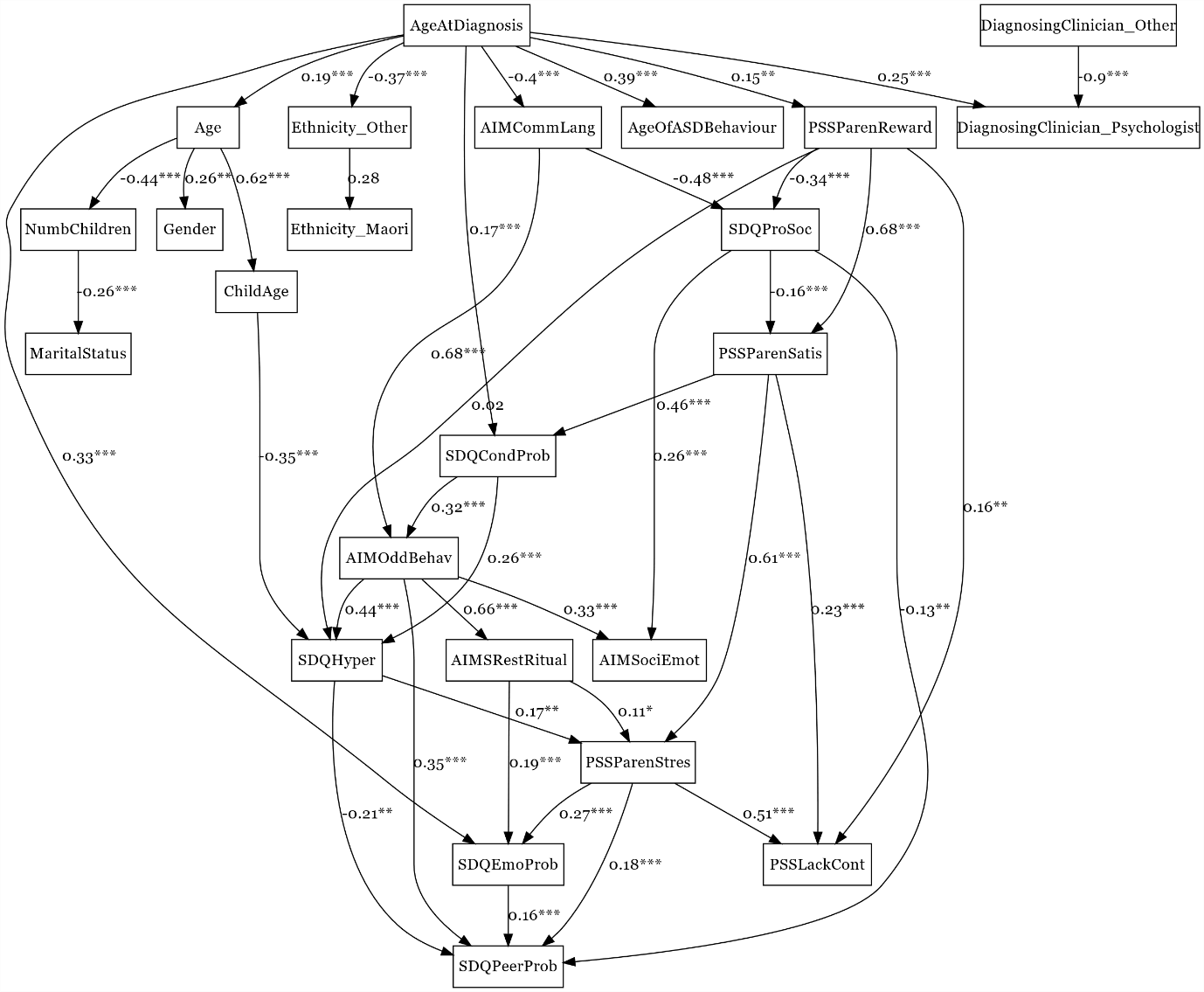


**Figure 8**

*Centrality statistics Bayesian Network With Blacklist excluding some demographics*

|  |  |  |
| --- | --- | --- |

**Table 1**

*Centrality statistics Variable Codes*

| Variable number | Short name | Variable name |
| --- | --- | --- |
| 1 | Ethnicity_Other | Ethnicity: Other |
| 2 | Age | Age |
| 3 | ChildAge | Child age |
| 4 | AgeAtDiagnosis | Age at Diagnosis |
| 5 | AIMSRestRitual | AIM: Restricted Ritualised Behaviour |
| 6 | AIMCommLang | AIM: Communication Language |
| 7 | AIMSociEmot | AIM: Socio-Emotional reciprocity |
| 8 | AIMODDBehav | AIM: Odd Behaviour |
| 9 | SDQCondProb | SDQ: Conduct Problems |
| 10 | SDQHyper | SDQ: Hyperactivity |
| 11 | SDQPeerProb | SDQ: Peer Problems |
| 12 | SDQProSoc | SDQ: Prosocial |
| 13 | SDQEmoProb | SDQ: Emotional Problems |
| 14 | PSSParenReward | PSS: Parental Reward |
| 15 | PSSParenStress | PSS: Parental Stress |
| 16 | PSSLackCont | PSS: Lack of Control |
| 17 | PSSParenSatis | PSS: Parental Satisfaction |

**R code for analysis**

library(missForest)

library(fastDummies)

## Set Working Directory

setwd("") ## Select file path

## Read Data and remove first row

dat.numb=read.csv("ASD_2023_Numbers.csv", header=T)

dat.numb.1=dat.numb[-c(1),]

## Replace empty cells with NA

dat.numb.1[dat.numb.1==""]=NA

## Identify and remove rows with all missing values

missing=which(rowSums(is.na(dat.numb.1))==ncol(dat.numb.1))

dat.numb2=dat.numb.1[-missing,]

## Select demographic variables and rename them

demo.numb=dat.numb2[,c(1:3, 5,7:12,14)] ## 11 variables

names(demo.numb)=c("Gender", "Age","Ethnicity", "Education" , "Marital_Status", "Child_Age", "Child_Gender", "No_Children", "Age_of_ASD_Behaviour", "Diagnosing_Clinician", "Age_At_Diagnosis")

## Select,order,and rename variables from the AIMS assessment

aims=dat.numb2[,c(15:27, 33:39, 28:32)] ## Kain 2014 4 compsite

for(i in 1:ncol(aims)){

names(aims)[i]=paste("aims", i, sep="_")

}

## Select,order,and rename variables from the SDQ assessment

sdq=dat.numb2[,c(42,47,54,62,44,65,52,56, 60, 41,50,53,59,63,46,51,64,57,61,40,43,49,55,58)] ## Goodman 1997 5 composites

for(i in 1:2){

names(sdq)[i]=paste("sdq", i, sep="_") ## Missing question 3

}

for(i in 3:24){

names(sdq)[i]=paste("sdq", i+1, sep="_") ## Missing question 3

}

## Select,order,and rename variables from the PSS assessment

pss=dat.numb2[,c(66,70,71,72,73,84,68,74,76,77,78,82,80,81,79,83)] ## Berry Jones 1995 factor ordering ## 4 composites

for(i in 1:ncol(pss)){

names(pss)[i]=paste("pss", i, sep="_")

}

## Combine the three assessments

dat.numb3=cbind.data.frame(aims, sdq, pss)

## Read second data file, remove the first row and replace empty cells with missing values

dat_txt=read.csv("ASD_2023_Text.csv", header=T)

dat.txt.1=dat_txt[-c(1),]

dat.txt.1[dat.txt.1==""]=NA

## Identify and remove rows with all missing values

missing=which(rowSums(is.na(dat.txt.1))==ncol(dat.txt.1))

dat.txt2=dat.txt.1[-missing,]

## Replace missing values with empty string

dat.txt2[is.na(dat.txt2)]=""

## Obtain text in column "other" and place it in the column with the question

dat.txt2$Q7[dat.txt2$Q7=="Other (please specify):"]=dat.txt2$Q7_6_TEXT[dat.txt2$Q7=="Other (please specify):"]

dat.txt2$Q8[dat.txt2$Q8=="Other (please specify):"]=dat.txt2$Q8_5_TEXT[dat.txt2$Q8=="Other (please specify):"]

dat.txt2$Q15[dat.txt2$Q15=="Other (please specify):"]=dat.txt2$Q15_5[dat.txt2$Q15=="Other (please specify):"]

## Recode ethnicity

dat.txt2$Q7[dat.txt2$Q7=="& samoan"]="Other"

dat.txt2$Q7[dat.txt2$Q7=="Fijian Indian "]="Other"

dat.txt2$Q7[dat.txt2$Q7=="SAMOAN 1/4"]="Other"

dat.txt2$Q7[dat.txt2$Q7=="American"]="European"

dat.txt2$Q7[dat.txt2$Q7=="British"]="European"

dat.txt2$Q7[dat.txt2$Q7=="Australian aboriginal"]="European"

dat.txt2$Q7[dat.txt2$Q7=="British "]="European"

dat.txt2$Q7[dat.txt2$Q7=="Dutch"]="European"

dat.txt2$Q7[dat.txt2$Q7=="Irish"]="European"

dat.txt2$Q7[dat.txt2$Q7=="Nz European"]="European"

dat.txt2$Q7[dat.txt2$Q7=="North American"]="European"

dat.txt2$Q7[dat.txt2$Q7=="Kiwi"]="European"

dat.txt2$Q7[dat.txt2$Q7=="Other European "]="European"

dat.txt2$Q7[dat.txt2$Q7=="Kiwi Caucasian (not European) "]="European"

dat.txt2$Q7[dat.txt2$Q7=="Other European "]="European"

dat.txt2$Q7[dat.txt2$Q7=="pakeha"]="European"

dat.txt2$Q7[dat.txt2$Q7=="Welsh"]="European"

dat.txt2$Q7[dat.txt2$Q7=="German"]="European"

dat.txt2$Q7[dat.txt2$Q7=="New Zealander"]="European"

dat.txt2$Q7[dat.txt2$Q7=="Nz European "]="European"

dat.txt2$Q7[dat.txt2$Q7=="Australian"]="European"

dat.txt2$Q7[dat.txt2$Q7=="Filipino"]="Asian"

dat.txt2$Q7[dat.txt2$Q7=="Filipino "]="Asian"

dat.txt2$Q7[dat.txt2$Q7=="Indian "]="Asian"

dat.txt2$Q7[dat.txt2$Q7=="Indian"]="Asian"

dat.txt2$Q7[dat.txt2$Q7=="Nepalese"]="Asian"

dat.txt2$Q7[dat.txt2$Q7=="Sri Lankan"]="Asian"

dat.txt2$Q7[dat.txt2$Q7=="half maori half european"]="Maori"

dat.txt2$Q7[dat.txt2$Q7=="Halfcaste maori/pakeha"]="Maori"

dat.txt2$Q7[dat.txt2$Q7=="Kiwi... part European, part Maori"]="Maori"

dat.txt2$Q7[dat.txt2$Q7=="Maori and pakeha"]="Maori"

dat.txt2$Q7[dat.txt2$Q7=="New Zealander of Maori and European descent not recognised in own country"]="Maori"

dat.txt2$Q7[dat.txt2$Q7=="Nz euro and nz Maori "]="Maori"

dat.txt2$Q7[dat.txt2$Q7=="This is not the correct way to ask an ethnicity question. I identify as M?ori and P?keh?"]="Maori"

dat.txt2$Q7[dat.txt2$Q7=="Maori/European"]="Maori"

dat.txt2$Q7[dat.txt2$Q7=="Middle Eastern/Latin American/African \n"]="Other"

dat.txt2$Q7[dat.txt2$Q7=="South African "]="Other"

dat.txt2$Q7[dat.txt2$Q7=="Pasifika"]="Other"

## Recode eduction

dat.txt2$Q8[dat.txt2$Q8=="Final year undergraduate studies, achieved fully to NCEA level 3"]="Secondary School"

dat.txt2$Q8[dat.txt2$Q8=="Certificate in dental assisting"]="Technical College or other professional training"

dat.txt2$Q8[dat.txt2$Q8=="Diploma"]="Technical College or other professional training"

dat.txt2$Q8[dat.txt2$Q8=="Diploma "]="Technical College or other professional training"

dat.txt2$Q8[dat.txt2$Q8=="NZQA LEVEL 3 AND 4 HOSPITALITY"]="Technical College or other professional training"

dat.txt2$Q8[dat.txt2$Q8=="University Undergraduate Diploma"]="Technical College or other professional training"

dat.txt2$Q8[dat.txt2$Q8=="Polytechnic "]="Technical College or other professional training"

dat.txt2$Q8[dat.txt2$Q8=="MBA"]="University Degree"

dat.txt2$Q8[dat.txt2$Q8=="MBA "]="University Degree"

dat.txt2$Q8[dat.txt2$Q8=="Master\x92s degree"]="University Degree"

dat.txt2$Q8[dat.txt2$Q8=="Grad diploma"]="University Degree"

dat.txt2$Q8[dat.txt2$Q8=="Honours degree"]="University Degree"

dat.txt2$Q8[dat.txt2$Q8=="I also have a Masters qualification"]="University Degree"

dat.txt2$Q8[dat.txt2$Q8=="Level 7 diploma"]="University Degree"

dat.txt2$Q8[dat.txt2$Q8=="MA"]="University Degree"

dat.txt2$Q8[dat.txt2$Q8=="Master in public health"]="University Degree"

dat.txt2$Q8[dat.txt2$Q8=="Masters"]="University Degree"

dat.txt2$Q8[dat.txt2$Q8=="Masters degree"]="University Degree"

dat.txt2$Q8[dat.txt2$Q8=="Masters Degree"]="University Degree"

dat.txt2$Q8[dat.txt2$Q8=="Masters of Arts in Education"]="University Degree"

dat.txt2$Q8[dat.txt2$Q8=="Mssters"]="University Degree"

dat.txt2$Q8[dat.txt2$Q8=="Pgc, pgd"]="University Degree"

dat.txt2$Q8[dat.txt2$Q8=="PhD"]="University Degree"

dat.txt2$Q8[dat.txt2$Q8=="Post-grad"]="University Degree"

dat.txt2$Q8[dat.txt2$Q8=="POST-GRADUATE"]="University Degree"

dat.txt2$Q8[dat.txt2$Q8=="post grad"]="University Degree"

dat.txt2$Q8[dat.txt2$Q8=="Post grad"]="University Degree"

dat.txt2$Q8[dat.txt2$Q8=="Post Grad"]="University Degree"

dat.txt2$Q8[dat.txt2$Q8=="Post grad medical "]="University Degree"

dat.txt2$Q8[dat.txt2$Q8=="post grad university"]="University Degree"

dat.txt2$Q8[dat.txt2$Q8=="Post graduate "]="University Degree"

dat.txt2$Q8[dat.txt2$Q8=="Post graduate degree"]="University Degree"

dat.txt2$Q8[dat.txt2$Q8=="Post graduate diploma"]="University Degree"

dat.txt2$Q8[dat.txt2$Q8=="Post Graduate Diploma"]="University Degree"

dat.txt2$Q8[dat.txt2$Q8=="Post graduate medical qualifications "]="University Degree"

dat.txt2$Q8[dat.txt2$Q8=="Postdoctoral"]="University Degree"

dat.txt2$Q8[dat.txt2$Q8=="Postgraduate"]="University Degree"

dat.txt2$Q8[dat.txt2$Q8=="Postgraduate certificate"]="University Degree"

dat.txt2$Q8[dat.txt2$Q8=="Primary School"]="Primary/Secondary School"

dat.txt2$Q8[dat.txt2$Q8=="Secondary School"]="Primary/Secondary School"

## Recode diagnosing clinician

dat.txt2$Q15[dat.txt2$Q15=="Private Psychologist"]="Psychologist"

dat.txt2$Q15[dat.txt2$Q15=="Consultant Clinical Psychologist at the hospital Child Development Service"]="Psychologist"

dat.txt2$Q15[dat.txt2$Q15=="Private Pediatrician"]="Pediatrician"

dat.txt2$Q15[dat.txt2$Q15==" Child Development Centre"]="Service/Multiple clinicians"

dat.txt2$Q15[dat.txt2$Q15=="Australian dx Paediatrician, SLT & Psychologist"]="Service/Multiple clinicians"

dat.txt2$Q15[dat.txt2$Q15=="CDC"]="Service/Multiple clinicians"

dat.txt2$Q15[dat.txt2$Q15=="CDC Waikato Hospital"]="Service/Multiple clinicians"

dat.txt2$Q15[dat.txt2$Q15=="CDS (NZ Child Development Service)"]="Service/Multiple clinicians"

dat.txt2$Q15[dat.txt2$Q15=="Chid Team Development Team, Puketiro - GP, Pediatrician, Clinical psychologist"]="Service/Multiple clinicians"

dat.txt2$Q15[dat.txt2$Q15=="Child and Adolescent uni"]="Service/Multiple clinicians"

dat.txt2$Q15[dat.txt2$Q15=="Child development assessment team at ccdhb"]="Service/Multiple clinicians"

dat.txt2$Q15[dat.txt2$Q15=="Child development service"]="Service/Multiple clinicians"

dat.txt2$Q15[dat.txt2$Q15=="Child Development Service"]="Service/Multiple clinicians"

dat.txt2$Q15[dat.txt2$Q15=="Child and Adolescent unit"]="Service/Multiple clinicians"

dat.txt2$Q15[dat.txt2$Q15=="Child development service - a panel of psychologist, pediatrician and speech therapist "]="Service/Multiple clinicians"

dat.txt2$Q15[dat.txt2$Q15=="Child development service - psychologist, paediatrician, speech therapist"]="Service/Multiple clinicians"

dat.txt2$Q15[dat.txt2$Q15=="Child development service - psychologist, paediatrician, speech therapist"]="Service/Multiple clinicians"

dat.txt2$Q15[dat.txt2$Q15=="It was a team with a pediatrician, psychologist and speech language therapist"]="Service/Multiple clinicians"

dat.txt2$Q15[dat.txt2$Q15=="Kindergarten"]="Service/Multiple clinicians"

dat.txt2$Q15[dat.txt2$Q15=="Private group, SLT, psychologist, physical therapist "]="Service/Multiple clinicians"

dat.txt2$Q15[dat.txt2$Q15=="MDAT Tauranga"]="Service/Multiple clinicians"

dat.txt2$Q15[dat.txt2$Q15=="My GP first noticed my daughter was ASD when she was 5 but it took till she was 7.5 for the child development team to formally diagnose her"]="Service/Multiple clinicians"

dat.txt2$Q15[dat.txt2$Q15=="Private group, SLT, psychologist, physical therapist"]="Service/Multiple clinicians"

dat.txt2$Q15[dat.txt2$Q15=="Vera Hayward Centre Dunedin"]="Service/Multiple clinicians"

dat.txt2$Q15[dat.txt2$Q15=="MDT"]="Service/Multiple clinicians"

dat.txt2$Q15[dat.txt2$Q15=="Waikids" ]="Service/Multiple clinicians"

dat.txt2$Q15[dat.txt2$Q15=="Whirinaki "]="Service/Multiple clinicians"

dat.txt2$Q15[dat.txt2$Q15=="Kindergarten "]="Service/Multiple clinicians"

dat.txt2$Q15[dat.txt2$Q15=="Psychiatrist "]="Psychiatrist"

dat.txt2$Q15[dat.txt2$Q15=="psychiatrist"]="Psychiatrist"

dat.txt2$Q15[dat.txt2$Q15=="Psychiatric"]="Psychiatrist"

dat.txt2$Q15[dat.txt2$Q15=="Child psychiatrist "]="Psychiatrist"

dat.txt2$Q15[dat.txt2$Q15=="Child Psychiatrist"]="Psychiatrist"

dat.txt2$Q15[dat.txt2$Q15=="Child psychiatrist"]="Psychiatrist"

dat.txt2$Q15[dat.txt2$Q15=="Paediatric Psychiatrist"]="Psychiatrist"

dat.txt2$Q15[dat.txt2$Q15=="Pediatric Psychiatrist"]="Psychiatrist"

dat.txt2$Q15[dat.txt2$Q15=="CAMHS psychiatrist"]="Psychiatrist"

dat.txt2$Q15[dat.txt2$Q15!="My Doctor/ General Practitioner (GP)"&dat.txt2$Q15!="My child has never received a formal diagnosis"&dat.txt2$Q15!="Pediatrician"&dat.txt2$Q15!="Psychologist"&dat.txt2$Q15!="Service/Multiple clinicians"&dat.txt2$Q15!="Psychiatrist"]="Other"

## Recode marital status

dat.txt2$Q9[dat.txt2$Q9=="In a relationship but living independently"]="Relationship"

dat.txt2$Q9[dat.txt2$Q9=="Married\n"]="Relationship"

dat.txt2$Q9[dat.txt2$Q9=="Married\n,In a relationship but living independently"]="Relationship"

dat.txt2$Q9[dat.txt2$Q9=="Married\n,Not married but living together/de facto relationship\n"]="Relationship"

dat.txt2$Q9[dat.txt2$Q9=="Married\n,Previously divorced"]="Relationship"

dat.txt2$Q9[dat.txt2$Q9=="Not married but living together/de facto relationship\n"]="Relationship"

dat.txt2$Q9[dat.txt2$Q9=="Previously divorced,In a relationship but living independently"]="Relationship"

dat.txt2$Q9[dat.txt2$Q9=="Previously divorced,Not married but living together/de facto relationship\n"]="Relationship"

dat.txt2$Q9[dat.txt2$Q9=="Previously divorced"]=""

dat.txt2$Q9[dat.txt2$Q9=="Single / a solo parent"]="Single"

dat.txt2$Q9[dat.txt2$Q9=="Single / a solo parent,Married\n" ]="Single"

dat.txt2$Q9[dat.txt2$Q9=="Single / a solo parent,Previously divorced"]="Single"

dat.txt2$Q9[dat.txt2$Q9=="Single / a solo parent,Previously divorced,In a relationship but living independently"]="Single"

## Select demographics and rename them

demo.txt2=dat.txt2[,c(1:3, 5,7:12,14)] ## 11 variables

names(demo.txt2)=c("Gender", "Age","Ethnicity", "Education" , "Marital_Status", "Child_Age", "Child_Gender", "No_Children", "Age_of_ASD_Behaviour", "Diagnosing_Clinician", "Age_At_Diagnosis")

## Replace right censor with maximum value

demo.txt2$Age[demo.txt2$Age=="70+"]=70

demo.txt2$Age=as.numeric(demo.txt2$Age)

demo.txt2$Child_Age[demo.txt2$Child_Age=="51+"]=51

demo.txt2$Child_Age=as.numeric(demo.txt2$Child_Age)

## Discretize age of parent

demo.txt2$Age=as.numeric(demo.txt2$Age)

demo.txt2$Age2=as.character(demo.txt2$Age)

demo.txt2$Age2[demo.txt2$Age<45]="<45"

demo.txt2$Age2[demo.txt2$Age>=45]="45+"

## Discretize age of child

demo.txt2$Child_Age=as.numeric(demo.txt2$Child_Age)

demo.txt2$Child_Age2=as.character(demo.txt2$Child_Age)

demo.txt2$Child_Age2[demo.txt2$Child_Age<13]="0-12"

demo.txt2$Child_Age2[demo.txt2$Child_Age>=13]="13+"

## Discretize number of children

demo.txt2$No_Children2=as.character(demo.txt2$No_Children)

demo.txt2$No_Children2[demo.txt2$No_Children=="None"]=0

demo.txt2$No_Children2[demo.txt2$No_Children=="1"|demo.txt2$No_Children=="2"]="1+"

demo.txt2$No_Children2[demo.txt2$No_Children=="3"|demo.txt2$No_Children=="4 or more"]="1+"

## Discretize age of ASD behaviour

demo.txt2$Age_of_ASD_Behaviour2=demo.txt2$Age_of_ASD_Behaviour

demo.txt2$Age_of_ASD_Behaviour2[demo.txt2$Age_of_ASD_Behaviour=="0-6 months"]="0-2"

demo.txt2$Age_of_ASD_Behaviour2[demo.txt2$Age_of_ASD_Behaviour=="6-12 months"]="0-2"

demo.txt2$Age_of_ASD_Behaviour2[demo.txt2$Age_of_ASD_Behaviour=="1 to 1.5 years"]="0-2"

demo.txt2$Age_of_ASD_Behaviour2[demo.txt2$Age_of_ASD_Behaviour=="1.5 - 2 years"]="0-2"

demo.txt2$Age_of_ASD_Behaviour2[demo.txt2$Age_of_ASD_Behaviour=="2 - 2.5 years"]="2+"

demo.txt2$Age_of_ASD_Behaviour2[demo.txt2$Age_of_ASD_Behaviour=="2.5 - 3 years"]="2+"

demo.txt2$Age_of_ASD_Behaviour2[demo.txt2$Age_of_ASD_Behaviour=="3 - 3.5 years"]="2+"

demo.txt2$Age_of_ASD_Behaviour2[demo.txt2$Age_of_ASD_Behaviour=="3.5 - 4 years"]="2+"

demo.txt2$Age_of_ASD_Behaviour2[demo.txt2$Age_of_ASD_Behaviour=="4 - 4.5 years"]="2+"

demo.txt2$Age_of_ASD_Behaviour2[demo.txt2$Age_of_ASD_Behaviour=="4.5 - 5 years"]="2+"

demo.txt2$Age_of_ASD_Behaviour2[demo.txt2$Age_of_ASD_Behaviour=="5 years or older"]="2+"

## Discretize age at diagnosis

demo.txt2$Age_At_Diagnosis2=demo.txt2$Age_At_Diagnosis

demo.txt2$Age_At_Diagnosis2[demo.txt2$Age_At_Diagnosis=="1 - 1.5 years"]="1-5"

demo.txt2$Age_At_Diagnosis2[demo.txt2$Age_At_Diagnosis=="1.5 - 2 years"]="1-5"

demo.txt2$Age_At_Diagnosis2[demo.txt2$Age_At_Diagnosis=="2 - 2.5 years"]="1-5"

demo.txt2$Age_At_Diagnosis2[demo.txt2$Age_At_Diagnosis=="2.5 - 3 years"]="1-5"

demo.txt2$Age_At_Diagnosis2[demo.txt2$Age_At_Diagnosis=="3 - 3.5 years"]="1-5"

demo.txt2$Age_At_Diagnosis2[demo.txt2$Age_At_Diagnosis=="3.5 - 4 years"]="1-5"

demo.txt2$Age_At_Diagnosis2[demo.txt2$Age_At_Diagnosis=="4 - 4.5 years"]="1-5"

demo.txt2$Age_At_Diagnosis2[demo.txt2$Age_At_Diagnosis=="4.5 - 5 years"]="1-5"

demo.txt2$Age_At_Diagnosis2[demo.txt2$Age_At_Diagnosis=="5 - 6 years"]="5+"

demo.txt2$Age_At_Diagnosis2[demo.txt2$Age_At_Diagnosis=="6 - 7 years"]="5+"

demo.txt2$Age_At_Diagnosis2[demo.txt2$Age_At_Diagnosis=="7 - 8 years"]="5+"

demo.txt2$Age_At_Diagnosis2[demo.txt2$Age_At_Diagnosis=="8 - 9 years"]="5+"

demo.txt2$Age_At_Diagnosis2[demo.txt2$Age_At_Diagnosis=="9 - 10 years"]="5+"

demo.txt2$Age_At_Diagnosis2[demo.txt2$Age_At_Diagnosis=="10 years or older"]="5+"

## Select new variables

demo.txt3=demo.txt2[,c(1,3:5,7,10,12:16)]

## Combine demographics and assessment data (Same row order, continuous variables recoded as discrete)

dat.numb5=cbind.data.frame(demo.txt3, dat.numb3)

## Change empty string to NA

dat.numb5[dat.numb5==""]=NA

## Change variables to numeric or factor variables

class1=vector()

for (i in 1: ncol(dat.numb5)){

class1[i]=class(dat.numb5[,i])

}

for (i in 12: ncol(dat.numb5)){

dat.numb5[,i]=as.numeric(dat.numb5[,i])

}

for(i in c(1:11)){

dat.numb5[,i]=as.factor(dat.numb5[,i])

}

## Reverse code PSS items

dat.numb5$pss_1=6-dat.numb5$pss_1

dat.numb5$pss_2=6-dat.numb5$pss_2

dat.numb5$pss_3=6-dat.numb5$pss_3

dat.numb5$pss_4=6-dat.numb5$pss_4

dat.numb5$pss_5=6-dat.numb5$pss_5

dat.numb5$pss_6=6-dat.numb5$pss_6

dat.numb5$pss_16=6-dat.numb5$pss_16

## Reverse code SDQ items

dat.numb5$sdq_7=(4-dat.numb5$sdq_7)

dat.numb5$sdq_14=(4-dat.numb5$sdq_14)

dat.numb5$sdq_15=(4-dat.numb5$sdq_15)

dat.numb5$sdq_17=(4-dat.numb5$sdq_17)

dat.numb5$sdq_18=(4-dat.numb5$sdq_18)

for(i in c(12:76)){

dat.numb5[,i]=as.factor(dat.numb5[,i])

}

## Remove participants with more than half of their data (assessment items) missing

missing2=which(rowSums(is.na(dat.numb5[,c(12:76)]))>ncol(dat.numb5[,c(12:76)])*.5)

dat.numb8=dat.numb5[-c(missing2),]

## Create Other level for demographics and recode them

dat.numb8$Ethnicity[dat.numb8$Ethnicity=="Asian"]="Other"

dat.numb8$Ethnicity=factor(dat.numb8$Ethnicity, levels=c("European","Maori","Other"))

dat.numb8$Diagnosing_Clinician[dat.numb8$Diagnosing_Clinician=="My child has never received a formal diagnosis"|dat.numb8$Diagnosing_Clinician=="My Doctor/ General Practitioner (GP)"|dat.numb8$Diagnosing_Clinician=="Psychiatrist"|dat.numb8$Diagnosing_Clinician=="Service/Multiple clinicians"]="Other"

dat.numb8$Diagnosing_Clinician=factor(dat.numb8$Diagnosing_Clinician, levels=c("Pediatrician","Psychologist", "Other"))

dat.numb8$Education=as.character(dat.numb8$Education)

## Create dummy variable for Education

dat.numb8$Education[dat.numb8$Education=="Primary/Secondary School"]="No University Degree"

dat.numb8$Education[dat.numb8$Education=="Technical College or other professional training"]="No University Degree"

## Change variable type

for (i in 1:ncol(dat.numb8)){

dat.numb8[,i]=as.factor(as.character(dat.numb8[,i]))

}

## Print dataset for descriptive Statistics

write.csv(dat.numb8, "Data_Before_Imputation.csv")

## Random forests imputation

set.seed(1000)

dat.numb9=missForest(dat.numb8)$ximp

## Change variable type

for(i in 12:ncol(dat.numb9)){

dat.numb9[,i]=as.numeric(as.character(dat.numb9[,i]))

}

## Calculate subscale scores

dat.numb9$AIMSRestRitual=rowSums(dat.numb9[,c(12:19)])

dat.numb9$AIMCommLang=rowSums(dat.numb9[,c(20:24)])

dat.numb9$AIMSociEmot=rowSums(dat.numb9[,c(25:31)])

dat.numb9$AIMOddBehav=rowSums(dat.numb9[,c(32:36)])

dat.numb9$SDQEmoProb=rowSums(dat.numb9[,c(37:40)]-1)

dat.numb9$SDQCondProb=rowSums(dat.numb9[,c(41:45)]-1)

dat.numb9$SDQHyper=rowSums(dat.numb9[,c(46:50)]-1)

dat.numb9$SDQPeerProb=rowSums(dat.numb9[,c(51:55)]-1)

dat.numb9$SDQProSoc=rowSums(dat.numb9[,c(56:60)]-1)

dat.numb9$PSSParenReward=rowSums(dat.numb9[,c(61:66)])

dat.numb9$PSSParenStres=rowSums(dat.numb9[,c(67:72)])

dat.numb9$PSSLackCont=rowSums(dat.numb9[,c(72:74)])

dat.numb9$PSSParenSatis=rowSums(dat.numb9[,c(66,75:76)])

## Select demographics and subscales only

dat.numb10=dat.numb9[,c(1:11, 77:89)]

## Recode demographics to dummy variables

dat.numb11=dummy_cols(dat.numb10, select_columns = c("Ethnicity","Diagnosing_Clinician"), remove_most_frequent_dummy = TRUE)

## Select required variables for BN and Save dataset

dat.numb12=dat.numb11[,c(1, 25,26,3:5,27,28,7:24)]

write.csv(dat.numb12, "Data_Ready_for_Analysis.csv")

## Clear environment

rm(list = ls(all.names = TRUE))

## Bayeisan network Statistics

library(bnlearn)

library(lavaan)

library(stats)

library(igraph)

library(lavaanPlot)

library(magick)

library(convertGraph)

library(networkD3)

library(Rgraphviz)

## Set Working Directory

setwd("") ## Select file path

## Read data

dat=read.csv("Data_Ready_for_Analysis.csv", header=T)[,c(2:27)]

## Change variable type

for(i in c(1:13)){

dat[,i]=as.factor(dat[,i])

}

for(i in c(14:ncol(dat))){

dat[,i]=as.numeric(dat[,i])

}

## Rename variables for BN

names(dat)=c("Gender","Ethnicity_Maori","Ethnicity_Other","Education","MaritalStatus","ChildGender","DiagnosingClinician_Other","DiagnosingClinician_Psychologist", "Age", "ChildAge","NumbChildren","AgeOfASDBehaviour","AgeAtDiagnosis","AIMSRestRitual","AIMCommLang","AIMSociEmot","AIMOddBehav","SDQCondProb", "SDQHyper","SDQPeerProb","SDQProSoc","PSSParenReward","PSSParenStres","PSSLackCont","PSSParenSatis","SDQEmoProb")

## Remove unconnected demographics for second network

dat2=dat[,-c(1,2,4,5,6,7,8,11,12)]

## Set Working Directory

setwd("") ## Select file path to results

## Write network blacklists

tiers <- list(names(dat)[c(1:21,26)], names(dat)[c(22:25)])

bl <- bnlearn::tiers2blacklist(tiers)

tiers2 <- list(names(dat2)[c(1:12, 17)], names(dat2)[c(13:16)])

bl2 <- bnlearn::tiers2blacklist(tiers2)

## Implement Bayesian networks

mod1=tabu(dat) ## No blacklist; All demographics

mod2=tabu(dat, blacklist = bl) ## Blacklist; All demographics

mod3=tabu(dat2) ## No blacklist; Without removed demographics

mod4=tabu(dat2, blacklist = bl2) ## Blacklist; Without removed demographics

## Obtain paramaters

param1=bn.fit(mod1, dat)

param2=bn.fit(mod2, dat)

param3=bn.fit(mod3, dat2)

param4=bn.fit(mod4, dat2)

## Transforming binary variables to ordinal variables for Lavaan

dat[,c("Gender","Ethnicity_Maori" ,"Ethnicity_Other", "Education","MaritalStatus","ChildGender","DiagnosingClinician_Other","DiagnosingClinician_Psychologist","Age","ChildAge","NumbChildren","AgeOfASDBehaviour","AgeAtDiagnosis")] <-

lapply(dat[,c("Gender","Ethnicity_Maori" ,"Ethnicity_Other", "Education","MaritalStatus","ChildGender","DiagnosingClinician_Other","DiagnosingClinician_Psychologist","Age","ChildAge","NumbChildren","AgeOfASDBehaviour","AgeAtDiagnosis")], ordered)

dat2[,c("Ethnicity_Other", "Age", "ChildAge","AgeAtDiagnosis")] <-

lapply(dat2[,c("Ethnicity_Other", "Age", "ChildAge","AgeAtDiagnosis")], ordered)

## Write SEM models for Lavaan

mod1

SEMmod1='

Ethnicity_Other~AgeAtDiagnosis

DiagnosingClinician_Psychologist~DiagnosingClinician_Other+AgeAtDiagnosis

Age~AgeAtDiagnosis

AgeOfASDBehaviour~AgeAtDiagnosis

AIMCommLang~AgeAtDiagnosis

PSSParenReward~AgeAtDiagnosis

Gender~Age

Ethnicity_Maori~Ethnicity_Other

ChildAge~Age

NumbChildren~Age

SDQProSoc~AIMCommLang+PSSParenReward

MaritalStatus~NumbChildren

PSSParenSatis~SDQProSoc+PSSParenReward

SDQCondProb~AgeAtDiagnosis+PSSParenSatis

AIMOddBehav~AIMCommLang+SDQCondProb

AIMSRestRitual~AIMOddBehav

AIMSociEmot~AIMOddBehav+SDQProSoc

SDQHyper~ChildAge+AIMOddBehav+SDQCondProb+PSSParenReward

PSSParenStres~AIMSRestRitual+SDQHyper+PSSParenSatis

PSSLackCont~PSSParenReward+PSSParenStres+PSSParenSatis

SDQEmoProb~AgeAtDiagnosis+AIMSRestRitual+PSSParenStres

SDQPeerProb~AIMOddBehav+SDQHyper+SDQProSoc+PSSParenStres+SDQEmoProb

'

mod2

SEMmod2='

Ethnicity_Other~AgeAtDiagnosis

DiagnosingClinician_Psychologist~DiagnosingClinician_Other+AgeAtDiagnosis

Age~AgeAtDiagnosis

AgeOfASDBehaviour~AgeAtDiagnosis

AIMCommLang~AgeAtDiagnosis

Gender~Age

Ethnicity_Maori~Ethnicity_Other

ChildAge~Age

NumbChildren~Age

AIMOddBehav~Ethnicity_Other+AIMCommLang

MaritalStatus~NumbChildren

AIMSRestRitual~AIMOddBehav

SDQHyper~ChildAge+AIMOddBehav

SDQCondProb~AgeAtDiagnosis+AIMOddBehav+SDQHyper

SDQProSoc~AIMCommLang+SDQCondProb

SDQEmoProb~AgeAtDiagnosis+AIMSRestRitual+SDQCondProb

AIMSociEmot~AIMOddBehav+SDQProSoc

SDQPeerProb~AIMOddBehav+SDQProSoc+SDQEmoProb

PSSParenReward~AIMCommLang+SDQCondProb+SDQProSoc+SDQEmoProb

PSSParenSatis~SDQCondProb+SDQHyper+SDQProSoc+PSSParenReward

PSSParenStres~AIMCommLang+SDQHyper+PSSParenSatis+SDQEmoProb

PSSLackCont~PSSParenReward+PSSParenStres+PSSParenSatis

'

mod3

SEMmod3='

AgeAtDiagnosis~Ethnicity_Other

Age~AgeAtDiagnosis

AIMCommLang~AgeAtDiagnosis

PSSParenReward~AgeAtDiagnosis

ChildAge~Age

SDQProSoc~AIMCommLang+PSSParenReward

PSSParenSatis~SDQProSoc+PSSParenReward

SDQCondProb~AgeAtDiagnosis+PSSParenSatis

AIMOddBehav~AIMCommLang+SDQCondProb

AIMSRestRitual~AIMOddBehav

AIMSociEmot~AIMOddBehav+SDQProSoc

SDQHyper~ChildAge+AIMOddBehav+SDQCondProb+PSSParenReward

PSSParenStres~AIMSRestRitual+SDQHyper+PSSParenSatis

PSSLackCont~PSSParenReward+PSSParenStres+PSSParenSatis

SDQEmoProb~AgeAtDiagnosis+AIMSRestRitual+PSSParenStres

SDQPeerProb~AIMOddBehav+SDQHyper+SDQProSoc+PSSParenStres+SDQEmoProb

'

mod4

SEMmod4='

Ethnicity_Other~AgeAtDiagnosis

Age~AgeAtDiagnosis

AIMCommLang~AgeAtDiagnosis

ChildAge~Age

AIMOddBehav~Ethnicity_Other+AIMCommLang

SDQHyper~ChildAge+AIMOddBehav

SDQCondProb~AgeAtDiagnosis+AIMOddBehav+SDQHyper

SDQProSoc~AIMCommLang+SDQCondProb

SDQEmoProb~AgeAtDiagnosis+SDQCondProb

AIMSRestRitual~AIMCommLang+AIMOddBehav+SDQEmoProb

AIMSociEmot~AIMOddBehav+SDQProSoc

SDQPeerProb~AIMOddBehav+SDQProSoc+SDQEmoProb

PSSParenReward~AIMCommLang+SDQCondProb+SDQProSoc+SDQEmoProb

PSSParenSatis~SDQCondProb+SDQHyper+SDQProSoc+PSSParenReward

PSSParenStres~AIMCommLang+SDQHyper+PSSParenSatis+SDQEmoProb

PSSLackCont~PSSParenReward+PSSParenStres+PSSParenSatis

'

## Implement SEM in Lavaan

sys1=sem(model=SEMmod1, data=dat, ordered = c("Gender","Ethnicity_Maori","Ethnicity_Other","Education","DiagnosingClinician_Other","DiagnosingClinician_Psychologist","Age","NumbChildren","AgeOfASDBehaviour","AgeAtDiagnosis"))

sys2=sem(model=SEMmod2, data=dat, ordered = c("Gender","Ethnicity_Maori","Ethnicity_Other","Education","DiagnosingClinician_Other","DiagnosingClinician_Psychologist","Age","NumbChildren","AgeOfASDBehaviour","AgeAtDiagnosis"))

sys3=sem(model=SEMmod3, data=dat2, ordered = c("Ethnicity_Other","Age","ChildAge","AgeAtDiagnosis"))

sys4=sem(model=SEMmod4, data=dat2, ordered = c("Ethnicity_Other","Age","ChildAge","AgeAtDiagnosis"))

## Set Working Directory for results

setwd("") ## Select file path

##Plot BN's

svg("BN_No_Blacklist_Demographics.svg")

g <- Rgraphviz::layoutGraph(bnlearn::as.graphNEL(mod1))

graph::nodeRenderInfo(g) <- list(fontsize=35)

Rgraphviz::renderGraph(g)

dev.off()

svg("BN_Blacklist_Demographics.svg")

g <- Rgraphviz::layoutGraph(bnlearn::as.graphNEL(mod2))

graph::nodeRenderInfo(g) <- list(fontsize=35)

Rgraphviz::renderGraph(g)

dev.off()

svg("BN_No_Blacklist_Removed_Demographics.svg")

g <- Rgraphviz::layoutGraph(bnlearn::as.graphNEL(mod3))

graph::nodeRenderInfo(g) <- list(fontsize=35)

Rgraphviz::renderGraph(g)

dev.off()

svg("BN_Blacklist_Removed_Demographics.svg")

g <- Rgraphviz::layoutGraph(bnlearn::as.graphNEL(mod4))

graph::nodeRenderInfo(g) <- list(fontsize=35)

Rgraphviz::renderGraph(g)

dev.off()

## Plot SEM with standardized coefficients

embed_plot_pdf(lavaanPlot(model=sys1, coefs=T, stars=c("regress"), stand = TRUE), "SEM_No_Blacklist_Demographics.pdf")

embed_plot_pdf(lavaanPlot(model=sys2, coefs=T, stars=c("regress"), stand = TRUE), "SEM_Blacklist_Demographics.pdf")

embed_plot_pdf(lavaanPlot(model=sys3, coefs=T, stars=c("regress"), stand = TRUE), "SEM_No_Blacklist_Demographics_Removed.pdf")

embed_plot_pdf(lavaanPlot(model=sys4, coefs=T, stars=c("regress"), stand = TRUE), "SEM_Blacklist_Demographics_Removed.pdf")

## Obtain fit statistics (BIC, AIC, Likelihood)

logLik(mod1, dat)## No blacklist; All demographics

logLik(mod2, dat)## Blacklist; All demographics

logLik(mod3, dat2)## No blacklist; Without removed demographics

logLik(mod4, dat2)## Blacklist; Without removed demographics

BIC(mod1, dat)

BIC(mod2, dat)

BIC(mod3, dat2)

BIC(mod4, dat2)

AIC(mod1, dat)

AIC(mod2, dat)

AIC(mod3, dat2)

AIC(mod4, dat2)

## Obtain fit statistics from SEM

fitmeasures(sys1)## No blacklist; All demographics

fitmeasures(sys2)## Blacklist; All demographics

fitmeasures(sys3)## No blacklist; Without removed demographics

fitmeasures(sys4)## Blacklist; Without removed demographics

## Obtain standardized closeness centrality metric for each network

close1=closeness(as.igraph(param1))

close1[is.nan(close1)]=NA

close1.1=(close1-mean(close1, na.rm=T))/sd(close1, na.rm=T)

close2=closeness(as.igraph(param2))

close2[is.nan(close2)]=NA

close2.1=(close2-mean(close2, na.rm=T))/sd(close2, na.rm=T)

close3=closeness(as.igraph(param3))

close3[is.nan(close3)]=NA

close3.1=(close3-mean(close3, na.rm=T))/sd(close3, na.rm=T)

close4=closeness(as.igraph(param4))

close4[is.nan(close4)]=NA

close4.1=(close4-mean(close4, na.rm=T))/sd(close4, na.rm=T)

## Obtain standardized Betweeness centrality metric for each network

between1=betweenness(as.igraph(param1))

between1[is.nan(between1)]=NA

between1.1=(between1-mean(between1, na.rm=T))/sd(between1, na.rm=T)

between2=betweenness(as.igraph(param2))

between2[is.nan(between2)]=NA

between2.1=(between2-mean(between2, na.rm=T))/sd(between2, na.rm=T)

between3=betweenness(as.igraph(param3))

between3[is.nan(between3)]=NA

between3.1=(between3-mean(between3, na.rm=T))/sd(between3, na.rm=T)

between4=betweenness(as.igraph(param4))

between4[is.nan(between4)]=NA

between4.1=(between4-mean(between4, na.rm=T))/sd(between4, na.rm=T)

## Obtain standardized degree centrality metric for each network

degree1=degree(as.igraph(param1), mode=c("all"))

degree2=degree(as.igraph(param2), mode=c("all"))

degree3=degree(as.igraph(param3), mode=c("all"))

degree4=degree(as.igraph(param4), mode=c("all"))

## Combine Centrality Statistics for each network

Central1=cbind.data.frame(Closeness=close1, Betweenness=between1, Degree=degree1)

Central2=cbind.data.frame(Closeness=close2, Betweenness=between2, Degree=degree2)

Central3=cbind.data.frame(Closeness=close3, Betweenness=between3, Degree=degree3)

Central4=cbind.data.frame(Closeness=close4, Betweenness=between4, Degree=degree4)

## Obtain probabilities from queries to the model

set.seed(1000)

cpquery(param4, event=(SDQCondProb>3), evidence = (AgeAtDiagnosis=="1-5"))

cpquery(param4, event=(SDQCondProb>3), evidence = (AgeAtDiagnosis=="5+"))

cpquery(param4, event=(AIMCommLang<14), evidence = (AgeAtDiagnosis=="1-5"))

cpquery(param4, event=(AIMCommLang<14), evidence = (AgeAtDiagnosis=="5+"))

cpquery(param4, event=(PSSParenStres>21), evidence = (AgeAtDiagnosis=="1-5"))

cpquery(param4, event=(PSSParenStres>21), evidence = (AgeAtDiagnosis=="5+"))

cpquery(param4, event=(SDQProSoc<3), evidence = (AgeAtDiagnosis=="1-5"))

cpquery(param4, event=(SDQProSoc<3), evidence = (AgeAtDiagnosis=="5+"))

cpquery(param4, event=(AIMCommLang<14), evidence = (PSSParenReward<13))

cpquery(param4, event=(AIMCommLang>=14), evidence = (PSSParenReward<13))

cpquery(param4, event=(AIMCommLang<14), evidence = (PSSParenStres>21))

cpquery(param4, event=(AIMCommLang>=14), evidence = (PSSParenStres>21))

cpquery(param4, event=(AIMCommLang<14), evidence = (PSSLackCont>8))

cpquery(param4, event=(AIMCommLang>=14), evidence = (PSSLackCont>8))

cpquery(param4, event=(AIMCommLang<14), evidence = (PSSParenSatis<8))

cpquery(param4, event=(AIMCommLang>=14), evidence = (PSSParenSatis<8))

## Clear environment

rm(list = ls(all.names = TRUE))

library(stringr)

library(bnlearn)

library(igraph)

library(dplyr)

## Set Working Directory

setwd("") ## Select file path

dat=read.csv("Data_Ready_for_Analysis.csv", header=T)[,c(2:27)]

## Change variable type

for(i in c(1:13)){

dat[,i]=as.factor(dat[,i])

}

for(i in c(14:ncol(dat))){

dat[,i]=as.numeric(dat[,i])

}

## Rename variables for BN

names(dat)=c("Gender","Ethnicity_Maori","Ethnicity_Other","Education","MaritalStatus","ChildGender","DiagnosingClinician_Other","DiagnosingClinician_Psychologist", "Age", "ChildAge","NumbChildren","AgeOfASDBehaviour","AgeAtDiagnosis","AIMSRestRitual","AIMCommLang","AIMSociEmot","AIMOddBehav","SDQCondProb", "SDQHyper","SDQPeerProb","SDQProSoc","PSSParenReward","PSSParenStres","PSSLackCont","PSSParenSatis","SDQEmoProb")

## Remove unconnected demographics for second network

dat2=dat[,-c(1,2,4,5,6,7,8,11,12)]

set.seed(9999)

## Write network blacklist

tiers2 <- list(names(dat2)[c(1:12, 17)], names(dat2)[c(13:16)])

bl2 <- bnlearn::tiers2blacklist(tiers2)

## Create datasets to fill

samp1=vector()

edge1.1=cbind.data.frame(edgefrom=NA,edgeto=NA, Samp=NA)

edge2.1=cbind.data.frame(edgefrom=NA,edgeto=NA, Samp=NA)

ParamCentral1.3=cbind.data.frame(Closeness=NA, Betweenness=NA, Degree=NA, samp=NA)

## Create column "row number"

dat2$X=seq(1,490,1)

## Obtain edges from networks on bootstrapped samples

for(i in 1:10000){

## Obtain random sample of row numbers

samp1=cbind.data.frame(X=sample(dat2$X, size=490, replace=T))

## Obtain bootstrapped dataset

datboot=merge(samp1, dat2, by="X", all.x=T)[,2:18]

## Implement hybrid BN

mod1.3=tabu(datboot, blacklist = bl2)

## Extract edges from BN

edge0.1=cbind.data.frame(edgefrom=mod1.3$arcs[,1], edgeto=mod1.3$arcs[,2], Samp=rep(i, nrow(mod1.3$arcs)))

##Combine edges from all bootstrapped networks

edge1.1=rbind.data.frame(edge1.1,edge0.1)

}

## Remove row of NA

edge1.2=edge1.1[-c(1),]

## Change variable type

edge1.2$Samp=as.numeric(edge1.2$Samp)

## Create vector of names

nodes2=names(dat2)[,2:18]

## Create empty list to fill

list1.1=list()

## Fill list with each BN

for(i in 1:10000){

arcs1.1=as.matrix(data.frame(from=edge1.2$edgefrom[edge1.2$Samp==i], to=edge1.2$edgeto[edge1.2$Samp==i]))

dag.1 <- empty.graph(nodes = nodes2)

arcs(dag.1) <- arcs1.1

name <- paste('BN:',i,sep='')

list1.1[[name]]=dag.1

}

## Compute edge strength and direction

Strength1.1=custom.strength(list1.1, nodes2)

## Construct averaged network

ave.bn1.1=averaged.network(Strength1.1)

## Set Working Directory for results

setwd("") ## Select file path

## Plot network

svg("Autism_Ave_BN_Blacklist_EmoPro.svg")

g <- Rgraphviz::layoutGraph(bnlearn::as.graphNEL(ave.bn1.1))

graph::nodeRenderInfo(g) <- list(fontsize=30)

Rgraphviz::renderGraph(g)

dev.off()
