# Supplementary material for: Factors predicting parenting stress in the autism spectrum disorder context: A network analysis approach
Source: PLoS One. 2025 Apr 21;20(4):e0319036. doi: 10.1371/journal.pone.0319036 (PMC12011239; doi:10.1371/journal.pone.0319036)
Supplement: Attachment — Submitted filename: Review Letter to the Editor.docx [file pone.0319036.s005.docx]

**Factors Predicting Parenting Stress in the Autism Spectrum Disorder Context: A Network Analysis Approach**

**Additional Questions:**
1. **Originality:** Does the paper contain new and significant information adequate to justify publication? NO

2. **Relationship to Literature:**  Does the paper demonstrate an adequate understanding of the relevant literature in the field and cite an appropriate range of literature sources? Is any significant work ignored? NO

3. **Methodology:**  Is the paper's argument built on an appropriate base of theory, concepts or other ideas?  Has the research or equivalent intellectual work on which the paper is based been well designed?  Are the methods employed appropriate? NO

4. **Results:**   Are results presented clearly and analyzed appropriately?  Do the conclusions adequately tie together the other elements of the paper? NO

5. **Practicality and/or Research implications:**

Implications for research, practice and/or society: Does the paper identify clearly any implications for research, practice and/or society? Does the paper bridge the gap between theory and practice? How can the research be used in practice (economic and commercial impact), in teaching, to influence public policy, in research (contributing to the body of knowledge)? What is the impact upon society (influencing public attitudes, affecting quality of life)? Are these implications consistent with the findings and conclusions of the paper? NO

6. **Quality of Communication:**  Does the paper clearly express its case, measured against the technical language of the fields and the expected knowledge of the journal's readership?  Has attention been paid to the clarity of expression and readability, such as sentence structure, jargon use, acronyms, etc. NO

**Confidential Comments to the Editor-in-Chief**

The author's details (name, department, university name, ORCID, correspondence email address, etc.) were given in this research article. Secondly, the quality of paper is not good as a whole. It wasted the time of the reviewers.

**Comments to the Author**

This paper, entitled “F**actors Predicting Parenting Stress in the Autism Spectrum Disorder Context: A Network Analysis Approach**," was submitted to PLOS ONE. It is not recommended for publication. Good luck for next time.
