# Supplementary material for: Factors predicting parenting stress in the autism spectrum disorder context: A network analysis approach
Source: PLoS One. 2025 Apr 21;20(4):e0319036. doi: 10.1371/journal.pone.0319036 (PMC12011239; doi:10.1371/journal.pone.0319036)
Supplement: Attachment — Submitted filename: Response to reviewers.docx [file pone.0319036.s006.docx]

Reviewer #1: Thank you very much for your invitation to review this exciting manuscript. After carefully reading this work, I indicate that this manuscript has merit and novelty. This manuscript is well-written, has a clear scope, and has high-standard scientific writing. Very good effort by the authors!

Introduction: This section is well-prepared and raises the research gap. Moreover, the authors indicate the novelty of their manuscript. The authors use appropriate references to describe the background. However, there are a few suggestions to enhance this manuscript regarding the missing essential references.

Hsiao, Y. J., Higgins, K., Pierce, T., Whitby, P. J. S., & Tandy, R. D. (2017). Parental stress, family quality of life, and family-teacher partnerships: Families of children with autism spectrum disorder. Research in developmental disabilities, 70, 152-162.

Papadopoulos A, Siafaka V, Tsapara A, et al. Measuring parental stress, illness perceptions, coping and quality of life in families of children newly diagnosed with autism spectrum disorder. BJPsych Open. 2023;9(3):e84. doi:10.1192/bjo.2023.55

Pisula, E., & Porębowicz-Dörsmann, A. (2017). Family functioning, parenting stress and quality of life in mothers and fathers of Polish children with high functioning autism or Asperger syndrome. PloS one, 12(10), e0186536.

Dijkstra-de Neijs, L., Boeke, D. B., van Berckelaer-Onnes, I. A., Swaab, H., & Ester, W. A. (2024). Parental Stress and Quality of Life in Parents of Young Children with Autism. Child Psychiatry & Human Development, 1-15.

Ilias, K., Cornish, K., Kummar, A. S., Park, M. S. A., & Golden, K. J. (2018). Parenting stress and resilience in parents of children with autism spectrum disorder (ASD) in Southeast Asia: A systematic review. Frontiers in psychology, 9, 280.

Papadopoulos, A., Fouska, S., Tafiadis, D., Trimmis, N., Plotas, P., & Siafaka, V. (2023). Psychometric Properties of the Greek Version of the Autism Parenting Stress Index (APSI) among Parents of Children with Autism Spectrum Disorder. Diagnostics, 13(20), 3259.

**Author Response. Thank you for bringing our attention to these papers. We have now weaved them into our manuscript’s Introduction and Discussion sections.**

Methodology:
The methodology is well-presented in all the subsections. The authors provide all the required information so other researchers can replicate this study.

Results: The authors detailed this section with many tables and figures.

Discussion:
The authors discussed the results of the study appropriately and adequately. They connect their findings with other studies. However, the authors should also add the above references suggested in their discussion.

**Author Response. Undertaken as directed.**

**We would like to thank Reviewer #1 for their suggestions to improve the manuscript.**

Reviewer #2: This paper, entitled “Factors Predicting Parenting Stress in the Autism Spectrum Disorder Context: A Network Analysis Approach," was submitted to PLOS ONE. It is not recommended for publication. Good luck for next time.

**Author Response. No response required.**

Reviewer #3: It is recommended to include the dataset in a public repository or as supplementary material to enhance transparency and reproducibility.

**Author Response. The R code and related data files have been added as Supplementary Materials.**

The sample's demographic bias (e.g., predominance of European mothers) limits generalizability. Discussing this limitation in more depth would strengthen the manuscript.

**Author Response. Additional content has been added to the Limitations section.**

**We would like to thank Reviewer #3 for their comments.**
